# Adjusting for BMI in analyses of volumetric mammographic density and breast cancer risk

**DOI:** 10.1186/s13058-018-1078-8

**Published:** 2018-12-29

**Authors:** Sue Hudson, Kirsti Vik Hjerkind, Sarah Vinnicombe, Steve Allen, Cassia Trewin, Giske Ursin, Isabel dos-Santos-Silva, Bianca L. De Stavola

**Affiliations:** 1 Department of Non-Communicable Disease Epidemiology, London School of Hygiene and Tropical Medicine, Keppel Street, London, WC1E 7HT UK; 2 Cancer Registry of Norway, Institute of Population-based Cancer Research, Oslo, Norway; 3 Division of Imaging and Technology, Ninewells Hospital Medical School, University of Dundee, Dundee, DD2 1SY UK; 4 Royal Marsden NHS Foundation Trust, London, SW3 6JJ UK; 5 Faculty of Population Health Sciences, Institute of Child Health, University College London, London, WC1N 1EH UK

**Keywords:** BMI, Breast cancer, Breast density, Mammographic density, OPERA

## Abstract

**Background:**

Fully automated assessment of mammographic density (MD), a biomarker of breast cancer risk, is being increasingly performed in screening settings. However, data on body mass index (BMI), a confounder of the MD–risk association, are not routinely collected at screening. We investigated whether the amount of fat in the breast, as captured by the amount of mammographic non-dense tissue seen on the mammographic image, can be used as a proxy for BMI when data on the latter are unavailable.

**Methods:**

Data from a UK case control study (numbers of cases/controls: 414/685) and a Norwegian cohort study (numbers of cases/non-cases: 657/61059), both with volumetric MD measurements (dense volume (DV), non-dense volume (NDV) and percent density (%MD)) from screening-age women, were analysed. BMI (self-reported) and NDV were taken as measures of adiposity. Correlations between BMI and NDV, %MD and DV were examined after log-transformation and adjustment for age, menopausal status and parity.

Logistic regression models were fitted to the UK study, and Cox regression models to the Norwegian study, to assess associations between MD and breast cancer risk, expressed as odds/hazard ratios per adjusted standard deviation (OPERA). Adjustments were first made for standard risk factors except BMI (minimally adjusted models) and then also for BMI or NDV. OPERA pooled relative risks (RRs) were estimated by fixed-effect models, and between-study heterogeneity was assessed by the *I*^2^ statistics.

**Results:**

BMI was positively correlated with NDV (adjusted *r* = 0.74 in the UK study and *r* = 0.72 in the Norwegian study) and with DV (*r* = 0.33 and *r* = 0.25, respectively). Both %MD and DV were positively associated with breast cancer risk in minimally adjusted models (pooled OPERA RR (95% confidence interval): 1.34 (1.25, 1.43) and 1.46 (1.36, 1.56), respectively; *I*^2^ = 0%, *P* >0.48 for both). Further adjustment for BMI or NDV strengthened the %MD–risk association (1.51 (1.41, 1.61); *I*^*2*^ = 0%, *P* = 0.33 and 1.51 (1.41, 1.61); *I*^2^ = 0%, *P* = 0.32, respectively). Adjusting for BMI or NDV marginally affected the magnitude of the DV–risk association (1.44 (1.34, 1.54); *I*^2^ = 0%, *P* = 0.87 and 1.49 (1.40, 1.60); *I*^2^ = 0%, *P* = 0.36, respectively).

**Conclusions:**

When volumetric MD–breast cancer risk associations are investigated, NDV can be used as a measure of adiposity when BMI data are unavailable.

## Introduction

Mammographic density captures the amount of (radio-)dense tissue in the breast. Mammographic density, for a woman’s age and body mass index (BMI), is a well-established breast cancer risk factor [1, 2]. This biomarker of risk is being increasingly used as an intermediate phenotype in epidemiological studies. It also offers the potential for breast cancer prevention strategies, including screening, to be tailored according to a woman’s individual risk, in combination with other non-genetic and genetic risk factors.

Mammographic density has traditionally been assessed as the absolute or relative amount (as percentage of the total breast size) occupied by the dense tissue which appears on a mammographic image as white “cotton-like” patches. Percent mammographic density (%MD) is negatively correlated with BMI (reported correlation coefficients ranging between −0.41 and −0.61 [3, 4]), which itself is a breast cancer risk factor (positively associated with risk in post-menopausal women but negatively associated with risk in pre-menopausal women) [5]. Therefore, it is essential to adjust for BMI (as well as age) and any study of mammographic percent density that fails to do so will lead to confounded estimates of the association between percent density and risk [6].

The recent introduction of full-field digital mammography (FFDM), paralleled by the development of fully automated digital image assessment software, has meant that mammographic density assessment is now routinely performed in many screening settings, thus providing a unique opportunity for the conduct of large-scale studies on this biomarker of risk. However, a common barrier to such investigations is the lack of information on a woman’s BMI as data on this variable are rarely collected at screening.

Most of the fully automated density assessment methods developed for FFDM attempt to estimate from the two-dimensional images, the volume of radio-dense tissue (DV) as well as the volume of non-dense (fat) tissue (NDV) and the total volume of the breast (BV) which, in Western populations, is highly correlated with NDV. Hence, NDV, or its BV correlate, has been used as a proxy for BMI in analyses of mammographic density and breast cancer risk in studies where BMI data are not available [7, 8]. However, the validity of such an approach has never been tested empirically. The aim of this study is to assess whether NDV can be used as a proxy for BMI when assessing associations between volumetric estimates of mammographic density, as derived from two-dimensional images, and breast cancer risk.

## Methods

### Study participants

The present analysis was conducted within two studies: a case control study from the UK and a cohort study from Norway.

#### UK study

The study methodology of the UK case control study is described in detail elsewhere [9]. In short, cases (*n* = 414) were women with newly diagnosed breast cancer at the Royal Marsden Hospital (RMH), London, between April 2010 and July 2012. Controls (*n* = 685) were women screened and found to be breast cancer-free at the Central and East London Breast Screening Service (CELBSS) in the same time period. The CELBSS invites women between 50 and 70 years of age for mammographic screening once every 3 years as part of the English National Health Service Breast Screening Programme. Women over 70 years can optionally contact the service for a self-referred appointment every 3 years.

Data on breast cancer risk factors, including age, ethnicity, parity, menopausal status, use of oral contraceptives, use of hormone therapy and self-reported height and weight, were collected by a self-administered questionnaire at the time of screening for controls and within 15.5 months of the diagnostic mammography for cases. BMI was calculated as weight in kg/(height in m)^2^. Ethnicity was categorised in accordance with the census classification as “White”, “Black” (African or Caribbean), “Asian” (Indian, Pakistani or Bangladeshi) and “Other” [10].

Participants underwent full-field digital mammography, with two views (cranio-caudal (CC) and mediolateral-oblique (MLO)), of both breasts. The images were taken on Senographe DS machines (GE Healthcare, Slough, UK). The anonymised raw images were analysed by using Volpara version 1.0 (Matakina Technology Limited, Wellington, New Zealand) [11]. This algorithm provided fully automated estimates of the volumes (all in cm^3^) of the total breast (BV), non-dense (fat) tissue (NDV) and dense (fibro-glandular) tissue (DV) separately for each one of the four breast/view images, and percent mammographic density (%MD) was estimated as DV/BV×100.

#### Norwegian study

The Cancer Registry of Norway is responsible for the administration of BreastScreen Norway (the Norwegian Breast Cancer Screening Program). All women within a targeted age-range of 50–69 years resident in the country are invited to undergo mammography screening every 2 years. From August 2006 to 2014, women who underwent mammographic screening in the nationwide programme were asked to complete a questionnaire on a number of standard breast cancer risk factors and a second questionnaire on current exposure to risk factors. Included in the present study were women who participated in BreastScreen Norway in four counties, had information on volumetric mammographic density from their first mammographic screening between 2007 and 2014, and had completed both questionnaires. However, for the second questionnaire on current exposures, if the questionnaire or certain values were missing, information from the questionnaire completed at a previous screening round was used (approximately 16.5%). The cohort consisted of 61,716 women, including 657 women who were diagnosed with a first occurrence of breast cancer during a median follow-up from date of screening of 3.84 (interquartile range 2.08, 4.83) years. Women with a previous diagnosis of breast cancer (*n* = 970), a ductal carcinoma *in situ* (DCIS) diagnosis up to 6 months after the screening date (*n* = 224) and a bilateral breast cancer (*n* = 11) were excluded.

In a similar manner to the UK study, all women had standard two-view full-field digital mammography of each breast with Senographe DS or Senographe Essential machines (GE Healthcare) or MDM L50 or MDM L30 machines (Phillips). The raw images were read by Volpara version 1.5.0 (Volpara Health Technologies Limited, Wellington, New Zealand) to obtain, similarly to the UK study, volumetric estimates of BV, NDV, DV and %MD.

### Ethical approval

The UK study was approved by all relevant ethics committees (Research Ethics Committees from the Royal Marsden Hospital, the Barts and the London NHS Trust, and the London School of Hygiene and Tropical Medicine). The Norwegian study was approved by the Regional Committee for Medical and Health Research ethics in the South-East Health Region of Norway. In the UK, participants provided written informed consent. In the Norwegian study, in accordance with the Cancer Registry Regulations, returning the questionnaire was considered consent, and information about screening examinations can be used for quality assurance and research if the women have not actively opted out. About 2% of the women attending the programme have opted out.

### Statistical methods

Descriptive analysis of UK controls and the full Norwegian cohort included examination of the distributions of BMI and volumetric mammographic measurements. For these analyses, measurements were averaged over the four images (that is, left and right CC and MLO images). Natural-log transformations were applied to average %MD, DV, NDV and BMI to normalise their distributions. Scatter plots and Pearson’s correlation coefficients were used to examine BMI associations with %MD, DV, NDV and BV. BMI and each mammographic measure were regressed on age at mammogram, parity and menopausal status using linear regression (including controls only in the UK study and the full cohort in the Norwegian study). Pearson’s correlation coefficients between the residuals derived from these models were then calculated (and denoted r) to allow examination of correlations that are not influenced by these variables.

For the UK case control analysis, the average density measures from the CC and MLO images from the unaffected breasts for cases and for a randomly selected breast for controls were used. In order to compare the association of %MD and DV with the odds of breast cancer, after adjusting for different sets of confounders, three different logistic regression models were fitted where these two exposures were first standardised as recommended previously [12]. The resulting estimates are referred to as OPERA ORs (“odds ratios per adjusted standard deviation”) and are effects per residual standard deviation of the exposure once its association with the confounders is accounted for. Estimation requires first fitting a linear regression model of the exposure on the confounders and then using the standardised residuals derived from this model as the exposure of interest in logistic regression models that include the same confounders. Fifty-one cases and 38 controls (8.1% of the study participants) were excluded from all logistic regression analyses because they were missing at least one of the variables used in the modelling.

The first (minimally adjusted) model controlled for age (continuous), menopausal status (pre-, peri/post-) and parity (yes/no). (Further adjustment for ethnicity, use of exogenous hormones and the other variables listed in Table 1 was also considered but it is not shown as it yielded similar results.) Second, a model was fitted that additionally adjusted for self-reported BMI. Finally, an alternative model was fitted that additionally adjusted for log-transformed NDV in place of BMI. Adjustment for BV instead of NDV was not considered because, albeit this variable is highly correlated with NDV (*r* = 0.99; *P* = 0.001), its interpretation is made more difficult by the fact that it reflects both DV and NDV.

**Table 1:** Baseline characteristics of the participants by status in the UK and Norwegian studies^a^

UK case control study			Norwegian cohort study	
	Controls (*n* = 685)	Cases (*n* = 414)	Non-cases (*n* = 61,059)	Cases (*n* = 657)
Age at mammography				
Mean (SD)	59.5 (6.6)	67.5 (12.7)	56.9 (5.74)	57.7 (5.43)
Number	679	412	61,059	657
BMI^b^				
Mean (SD)	26.1 (5.6)	26.4 (4.9)	25.6 (4.2)	25.8 (4.1)
Number	656	368	54,345	589
Ethnicity (UK)/Country of birth (Norway), n (%)				
White/Norway	520 (76.5)	370 (89.4)	56,234 (93.8)	612 (94.2)
Non-white/Outside Norway	160 (23.5)	39 (9.6)	3693 (6.2)	38 (5.8)
Missing	5	5	1132	7
Family history of BC, n (%)				
No	N/A	N/A	45,168 (77.1)	447 (70.0)
Yes	N/A	N/A	13,390 (22.9)	192 (30.0)
Missing			2501	18
Menopausal status^c^, n (%)				
Pre- + peri-menopausal	91 (13.3)	55 (13.3)	14,776 (25.2)	141 (22.1)
Post-menopausal	591 (86.7)	358 (86.7)	43,856 (74.8)	496 (77.9)
Missing	3	1	2427	20
Parity, n (%)				
Nulliparous	209 (30.9)	65 (15.9)	4946 (8.5)	57 (9.0)
Parous	467 (69.1)	343 (84.1)	53,563 (91.5)	577 (91.0)
Missing	9	6	2550	23
Age at menarche in years, n (%)				
<13	271 (53.9)	159 (54.1)	16,764 (40.9)	186 (41.9)
14+	232 (46.1)	135 (45.9)	24,202 (59.1)	258 (58.1)
Missing	14	33	4107	43
Hormone therapy use, n (%)				
No	459 (68.8)	246 (63.2)	34,150 (66.2)	305 (55.6)
Yes	208 (31.2)	143 (36.8)	17,418 (33.8)	244 (44.4)
Missing	18	25	9491	108
Educational level, n (%)				
None/primary school	35 (5.2)	17 (6.2)		
Lower secondary			13,772 (23.3)	164 (25.9)
Secondary or higher	641 (94.8)	225 (93.8)	45,457 (76.7)	470 (74.1)
Missing	9	142	1830	23
Breastfeeding among parous women, n (%)				
Yes	358 (76.7)	224 (74.7)	46,107 (99.9)	497 (100)
Missing	3	43	9929	103

Abbreviations: *BC* breast cancer, *BMI* body mass index, *N/A* data not available, *SD* standard deviation

^a^Percentages calculated without missing values

^b^BMI estimated from self-reported height and weight as weight/height^2^ (in kg/m^2^)

^c^Post-menopausal women defined as those who self-reported natural (cessation of menses for at least 12 months) or surgical menopause, were older than 55 years, or had ever used hormone therapy. Owing to small numbers, pre-menopausal (younger than 55 years and still having regular periods) and peri-menopausal (younger than 55 years and having irregular periods) women were combined into a single category

In the Norwegian cohort study, average density measures were based on log-transformed average values of the CC and MLO readings from the unaffected breast for cases and from a randomly selected breast for non-cases. Cox regression proportional hazards models were fitted to the cohort data, using age as the time-scale, to evaluate the associations of (log-transformed and standardised as described above for the UK study) %MD and DV with breast cancer risk, expressed in terms of hazard ratios and referred to as OPERA HRs.

Three different models were fitted as in the UK study; the first was minimally adjusted for screening year (categorised using 2-year intervals), menopausal status (pre-, peri-, post-) and parity (yes/no) (further adjustment for country of birth as a proxy for ethnicity did not affect the findings). A second model was additionally adjusted for BMI, and a third model was additionally adjusted for NDV in place of BMI. In all, 10,288 participants, including 99 cases, were excluded from all three models because they missed data for at least one of the variables listed.

Three further models were also fitted to the Norwegian data using the full reproductive and lifestyle risk factor questionnaire data collected in this study (that is, screening year category, menopausal status, parity, age at menopause, age at menarche, age at first birth, duration of breastfeeding, use of hormone therapy, family history of breast cancer, education, smoking, alcohol use and physical activity level). In the first model, BMI was omitted; in the second model, BMI was included; in the third model, NDV was used instead of BMI. In total, 25,833 (41.9% of the original cohort) women with missing data on any of the variables examined were excluded to ensure that these additional models were fitted to the same subset of women. Departure from the proportional hazards assumption underlying each of these fitted models was evaluated by using tests based on Schoenfeld residuals. The Akaike information criterion (AIC) corresponding to each multivariable model from the two countries is also reported.

Similar analytical steps were followed to study the associations between BMI and breast cancer risk, and then NDV and breast cancer risk, in both studies, in each case adjusting for age, menopausal status and parity.

Fixed-effects models were used to obtain pooled summary OPERA relative risk (RR) estimates from the two studies. Between-study heterogeneity was assessed by the Q statistic and the *I*^2^ statistic [13].

In all the analyses, we considered statistical significance (two-sided) at a *P* value of less than 0.05. All analyses were conducted in Stata (IC 14 for the statistical analysis of the UK data and the meta-analysis and IC 15 for the analysis of the Norwegian data) [14].

## Results

### Study participants

The baseline characteristics of the participants in the two studies are shown in Table 1. In the UK, study cases were, on average, older than controls and more likely to be White. Likewise, cases were slightly older at mammography than non-cases in the Norwegian study. The mean BMI was similar for UK cases and controls and for Norwegian cases and non-cases.

### Correlations between BMI and volumetric mammographic measures

**Fig. 1:**
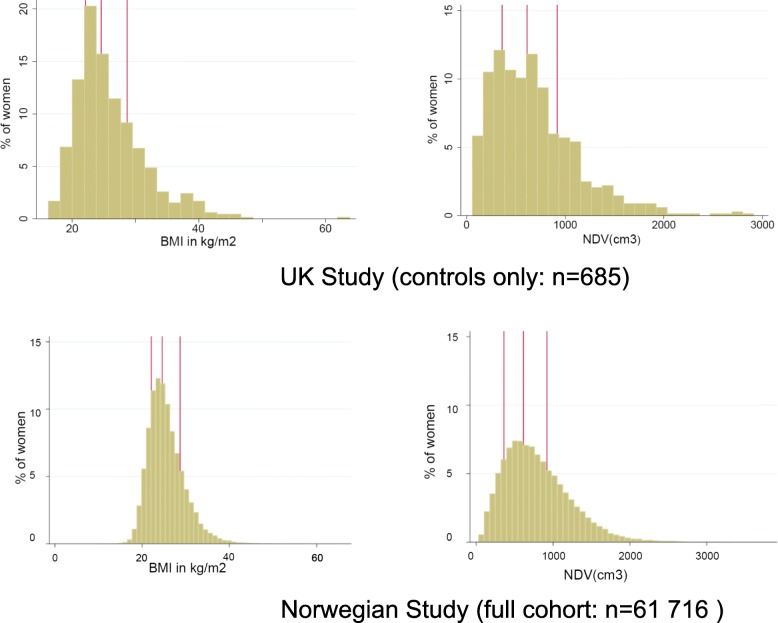
Distribution of self-reported BMI and measurements of volume of mammographic non-dense tissue in the UK and Norwegian studies. Abbreviations: *BMI* body mass index, *NDV* volume of mammographic non-dense tissue averaged over the cranio-caudal and mediolateral-oblique views from the left and right breasts. Vertical lines represent the median and interquartile range values

The distributions of self-reported BMI and of NDV, the volumetric measurement that reflects the fatty tissue in the breast, were right-skewed in the UK control group and in the full Norwegian cohort (Fig. 1). Table 2 shows the correlations between each volumetric measure and BMI after adjusting for age, parity and menopausal status. Notably, the two studies yielded very similar results. NDV was highly positively correlated with BMI in both the UK (*r* = 0.74) and in the Norwegian (*r* = 0.72) study. In contrast, the correlation between DV and BMI was weakly positive in both the UK study (*r* = 0.33) and the Norwegian study (*r* = 0.25). Consequently, %MD was negatively correlated with BMI in both the UK (*r* = −0.66) and Norwegian (*r* = −0.57) studies. The correlation between %MD and DV was only moderate after adjustment for age and BMI in the UK (*r* = 0.33) and Norwegian (*r* = 0.55) studies (data not shown). Further analyses showed that the findings were robust after stratification by mammographic view, age at mammography and, for the UK study, restricting the analysis to White women (data not shown).

**Table 2:** Pearson’s correlation coefficients of mammographic measures^a^ with BMI^a^ and with log NDV

	Adjusted log BMI		Adjusted log NDV	
	UK case control study controls only^b^	Norwegian cohort study full cohort^c^	UK case control study controls only^b^	Norwegian cohort study full cohort^c^
Adjusted log NDV	0.74	0.72	–	–
Adjusted log %MD	−0.66	−0.57	−0.80	−0.72
Adjusted log DV	0.33	0.25	0.57	0.43

Abbreviations: *BMI* body mass index, *DV* volume of mammographically dense tissue, *NDV* volume of mammographically non-dense tissue, *%MD* percent mammographic density

DV, NDV and %MD averaged over the cranio-caudal and mediolateral-oblique views from the left and right breasts

^a^All mammographic features as well as BMI were regressed on age at mammogram, parity and menopausal status and the residuals from these regressions were used to calculate the correlation coefficients and referred to as “adjusted” measures

^b^*n* = 646 (women with missing BMI, age, parity, menopausal status or mammographic measurements were excluded and one woman with a BMI greater than 60 was also excluded)

^c^*n* = 51,427 (women with missing BMI, age, parity, menopausal status or mammographic measurements were excluded or BMI greater than 60 were excluded)

*P* <0.0001 in all cases

### Associations between adiposity measures and breast cancer risk

There were weak positive associations between BMI and breast cancer risk (adjusted for age, menopausal status and parity) in both the UK (OPERA OR 1.10, 95% confidence interval (CI) 0.95, 1.26) and the Norwegian (OPERA HR 1.09, 95% CI 1.01, 1.19) studies. The magnitude of the BMI–risk association was not modified by menopausal status or age in either study (*P* > 0.30 and *P* > 0.10, respectively, in models that included interactions with either menopausal status or age), most likely because of the relatively small number of younger (pre-menopausal) women in either study. There was no evidence of an association between NDV and breast cancer risk adjusting for the same covariates as for BMI (UK study OPERA OR 0.96, 95% CI 0.83, 1.11); Norwegian study OPERA HR 1.01, 95% CI 0.93, 1.09).

### Associations between relative and absolute volumetric density and breast cancer risk

**Fig. 2:**
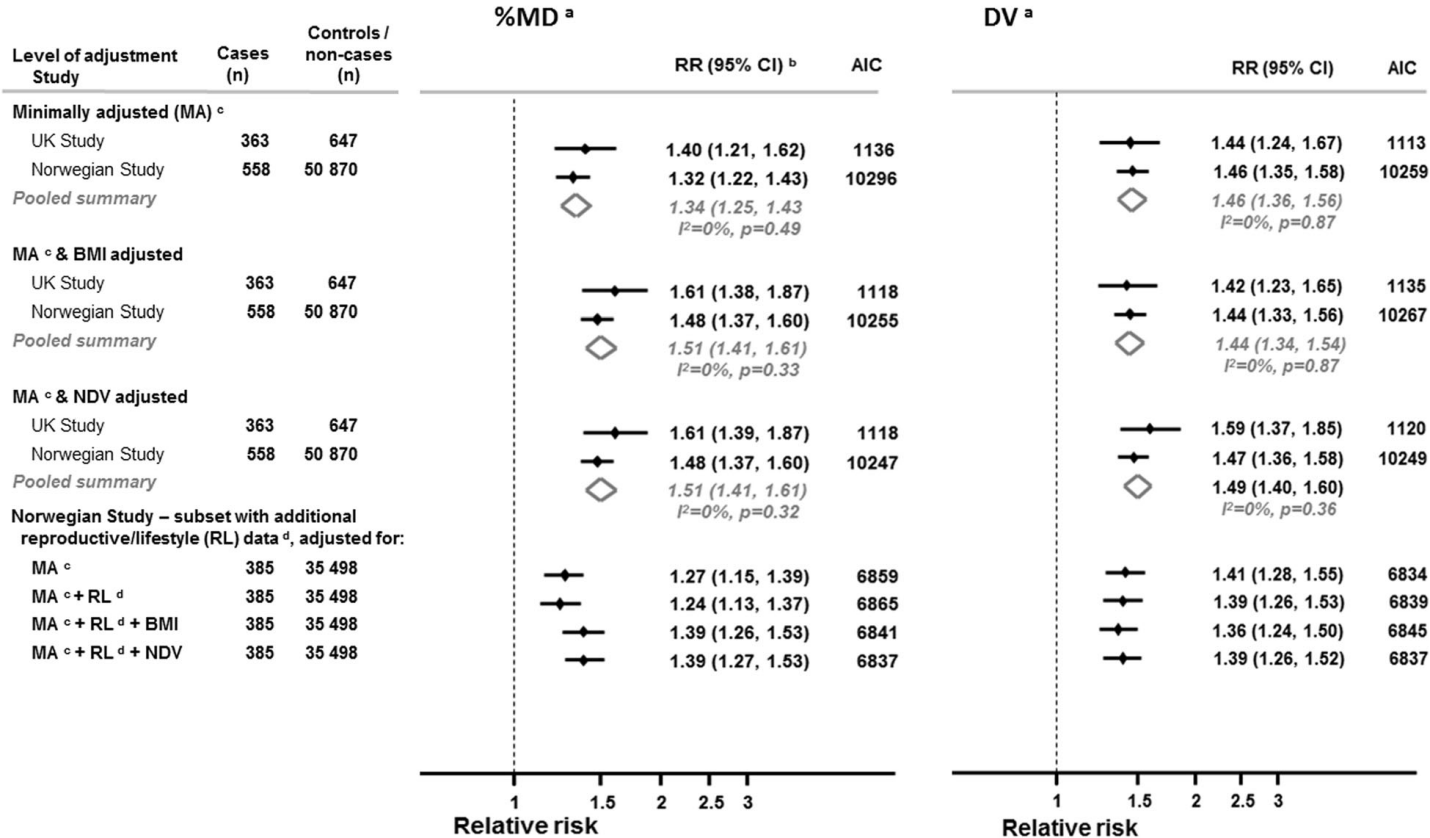
Mammographic density associations with breast cancer risk with and without adjustment for adiposity in the UK and Norwegian studies. Abbreviations: *BMI* body mass index, *CI* confidence interval, *DV* volume of mammographic dense tissue, *NDV* volume of mammographic non-dense (fat) tissue, *%MD* percent mammographic density. ^a^DV, NDV and %MD values are the average from the cranio-caudal and mediolateral-oblique views from the unaffected breast for cases and for a randomly selected breast side for controls, log-transformed. ^b^In the UK study, OPERA odds ratios (ORs) were estimated by a logistic regression. In the Norwegian cohort study, OPERA hazard ratios (HRs) were estimated by a Cox regression model in which attained age was taken as the time scale (see Methods section). ^c^Minimally adjusted model: analysis adjusted for age, menopausal status and parity in the UK study; analysis adjusted for screening year, menopausal status and parity (see Methods section). ^d^Model additionally adjusted for age at menopause, age at menarche, age at first birth, duration of breastfeeding, use of hormone therapy, family history of breast cancer, education, smoking, alcohol use and physical activity level

Figure 2 shows study-specific and pooled summary OPERA estimates of, respectively, %MD and DV with breast cancer risk. In both the UK and Norwegian studies, the minimally adjusted models, which exclude any adjustment for adiposity, show a positive association between %MD and breast cancer risk with no evidence of between-study heterogeneity (*I*^2^ = 0%; *P* = 0.49). Further adjustment for BMI showed a strengthening of the positive association between %MD and breast cancer risk: the magnitude of the pooled OPERA RR increased from 1.34 (95% CI 1.25, 1.43) to 1.51 (95% CI 1.41, 1.61) upon adjustment for BMI and there was no evidence of between-study heterogeneity for the latter (*I*^*2*^ = 0%; *P* = 0.33). Replacing BMI with NDV, as a proxy for level of adiposity, yielded the same strength of association between %MD and breast cancer risk with the pooled OPERA RR increasing to 1.51 (95% CI 1.41, 1.61) and there was no evidence of between-study heterogeneity (*I*^*2*^ = 0%; *P* = 0.33).

The association between DV and breast cancer risk was slightly stronger than that found for %MD in the minimally adjusted model (pooled OPERA RR for DV = 1.46, 95% CI 1.36, 1.56) and there was no evidence of between-study heterogeneity (*I*^*2*^ = 0%; *P* = 0.87). Adjusting for BMI had little impact on the magnitude of the DV–breast cancer risk association (pooled OPERA RR = 1.44, 95% CI 1.34, 1.54). When BMI was replaced by NDV, the magnitude of the pooled OPERA RR increased only slightly to 1.49 (95% CI 1.40, 1.60) and there was no evidence of between-study heterogeneity (*I*^*2*^ = 0%; *P* = 0.36).

The similarity of the estimated RRs for %MD and DV when adjusted for either BMI or NDV indicates that these measures of adiposity lead to equivalent control of confounding. Since the models adjusted for age, menopausal status, parity and NDV have the smallest AIC in both the UK and Norwegian studies, controlling for the NDV, when BMI is self-reported as in these datasets, appears to be (marginally) preferable.

Further analyses of the OPERA estimates in the Norwegian data show that the magnitude of the associations of %MD and DV with breast cancer risk was little changed by adjustment for additional reproductive and lifestyle factors in the subset of women with information on these variables. The addition of BMI to this expanded model strengthened the association between %MD and breast cancer risk; the OPERA HR increased from 1.24 (95% CI 1.13, 1.37) to 1.39 (95% CI 1.26, 1.53). Likewise, replacing BMI with NDV to control for adiposity led to similar estimates (OPERA HR = 1.39, 95% CI 1.27, 1.53) though with a marginally better fitting model using AIC. In contrast, adjustment for BMI or NDV had little effect on the magnitude of the DV–breast cancer risk association (Fig. 2).

## Discussion

### Main findings

We found that, for screening-aged women, the association between volumetric %MD and breast cancer risk is partly confounded by levels of adiposity and that the two measures of adiposity available in our studies—BMI or NDV—lead to similar adjusted estimates of association. In contrast, when assessing the magnitude of the association between volumetric absolute mammographic density (that is, DV) and breast cancer risk, adjustment for BMI or NDV had little or no impact on the magnitude of the association. All of these estimates of association are expressed in terms of units per relative standard deviation of the exposure (that is, using the OPERA approach). This allows us to compare estimates while adjusting for different sets of potential confounders, in units that account for the strength of association between the confounders and the exposure of interest [12]. Both the Norwegian cohort and the UK case control analyses found that it is important to adjust for adiposity when the main explanatory variable is volumetric %MD, as estimated from a two-dimensional image; otherwise, the relationship between %MD and breast cancer risk may appear to be weaker. Our results based on AIC suggest that objectively measured NDV may offer a slightly better proxy for adiposity than BMI when comparing the model’s specifications in terms of goodness of fit. This may be a consequence of the self-reported nature of the available BMI data, and measurement error may lead to attenuation of the adjustment. Nevertheless, it is unclear the extent to which BMI and NDV capture the same, or different, underlying biological entities.

After adjustment for age, parity and menopausal status, BMI was found to be strongly positively correlated with NDV but strongly negatively correlated with %MD. In contrast, BMI was weakly positively correlated with DV. The observed strong positive BMI–NDV correlation is consistent with findings from area-based mammographic studies [4, 6, 15]. The observed weak positive BMI–DV correlation is also in line with findings from other volumetric density studies [16, 17] but in contrast to those from area-based studies which consistently report a negative correlation [4, 18, 19]. The correlation between DV and %MD after adjustment for age and BMI was not as strong (in either the UK or Norwegian study) as that reported in area-based studies [20].

The present study found a positive, albeit weak, association between BMI and breast cancer risk, reflecting the predominantly peri-/post-menopausal status of the participants, but no association between NDV and breast cancer risk. There is little evidence for an NDV–breast cancer association from volumetric studies to date, but a meta-analysis of data from 13 area-based studies has reported an overall inverse association between mammographic non-dense area and risk [21] albeit with considerable between-study heterogeneity.

### Strengths and limitations

Strengths of this investigation include the availability of data from two independent studies of women of screening age. Both studies used the same objective volumetric density assessment method, making the two datasets comparable. In addition, the Norwegian study was population-based and had a very large sample size and detailed data on a wide range of potential confounding variables collected prior to breast cancer diagnosis and therefore was unlikely to have been affected by recall bias. Furthermore, although other studies have assumed that it is reasonable to used NDV as a surrogate for BMI [7, 8], we believe that we are the first to have formally tested this empirically.

A limitation of this investigation is that it relied on self-reported BMI. Previous research suggests that women tend to understate their weight and overstate their height, particularly those who are overweight or obese [22, 23], although a recent study found that women attending BreastScreen Norway reported weight and height within 1 kg/cm of directly measured values [24]. In most population-based screening programmes, however, it is logistically impossible to perform anthropometric measurements when women attend screening. Nevertheless, it would be informative if similar analyses were replicated within a study sample with measured BMI.

We used the OPERA approach to allow comparison across different exposures (that is, effects per residual standard deviation of the exposure once its association with the confounders is accounted for). It is argued that this provides a fairer comparison of the different risk gradients across the different models [12]. However, a common criticism of two-step approaches, such as OPERA, is that the standard errors of the estimated coefficients are underestimated and thus lead to a spurious increase in the precision of the estimated effect sizes [25].

The study was restricted to women of screening age and not generalizable to younger women. There is also evidence that the relationship between percent body fat and BMI is dependent upon ethnicity [26, 27], with Asians having a higher percentage of body fat for any given BMI compared with Caucasians [28]. The relatively small number of non-White women in both studies precluded examination by ethnicity.

Finally, both studies were based on a particular volumetric mammographic density assessment approach. It would be worthwhile to examine the extent to which the present findings can be replicated when alternative methods of assessment of volumetric mammographic density are used.

## Conclusions

The availability of fully automated methods to measure mammographic density enables the integration of such measurements within screening programme settings, thus facilitating the conduct of large-scale studies, including research on whether screening should be tailored to a woman’s individual risk. A perceived barrier to the conduct of such studies is the lack of information on a woman’s BMI. This study shows that the association between DV and breast cancer risk is not confounded by BMI or NDV and hence no adjustment for these variables is required. In contrast, the association between volumetric %MD and risk is confounded by level of adiposity and adjustment for either BMI or NDV yields similar results. Adjustment for NDV may offer some advantages over BMI as the NDV measurements are objective, being generated by a fully automated algorithm, and thus do not suffer from measurement errors associated with self-reported BMI. Furthermore, in most breast screening settings, it is not feasible to collect BMI data; therefore, NDV values are potentially very valuable because they will be automatically available for every woman screened. Nevertheless, these findings need to be replicated in other populations, particularly among those with a different age and ethnic mix.

## Abbreviations

%MD: Percentage mammographic density
AIC: Akaike information criterion
BMI: Body mass index
BV: Breast volume as ascertained from a mammogram
CC: Cranio-caudal view
CELBSS: Central and East London Breast Screening Service
CI: Confidence interval
DV: Dense volume (that is, absolute volume of (radio-)dense tissue seen on a mammogram)
FFDM: Full-field digital mammography
HR: Hazard ratio
MLO: Mediolateral oblique mammogram view
NDV: Non-dense volume (that is, volume of non-dense (fat) tissue seen on a mammogram)
OPERA: Odds per adjusted standard deviation
OR: Odds ratio
RR: Relative risk
